# Prognostic impact of collateral circulation in direct thrombectomy versus bridging thrombectomy for acute ischemic stroke patients with anterior circulation large vessel occlusion: a retrospective comparative study

**DOI:** 10.3389/fnins.2025.1624284

**Published:** 2025-09-11

**Authors:** Zuopeng Li, Chao Cai, Yuan Qin

**Affiliations:** ^1^The First Hospital of Shanxi Medical University, Taiyuan, China; ^2^The First Clinical Medical College of Shanxi Medical University, Taiyuan, China; ^3^Department of Neurology, The People's Hospital of Pianguan County, Pianguan, China

**Keywords:** acute ischemic stroke, thrombectomy, collateral circulation, anterior circulation occlusion, large vessel occlusion (LVO)

## Abstract

**Objective:**

To evaluate the prognostic impact of collateral circulation on outcomes of direct thrombectomy (DT) versus bridging thrombectomy (BT) for acute ischemic stroke (AIS) patients with anterior circulation large vessel occlusion (LVO).

**Method:**

This retrospective study included 460 AIS patients with anterior circulation LVO who underwent either DT (*n* = 220) or BT (*n* = 240). Collateral status was assessed using multiphase computed tomographic angiography. The primary outcome was functional prognosis at 3 months, measured by the modified Rankin Scale (mRS). Prognostic factors for good prognosis (90-day mRS ≤ 2) and adverse events were identified through multivariate logistic regression and receiver operating characteristic (ROC) analyses.

**Results:**

The BT group had significantly higher rates of intracerebral hemorrhage and hemorrhagic transformation compared to the DT group (*p* < 0.05). Good collateral circulation was independently associated with good prognosis and lower risk of serious adverse events (*p* < 0.001). Lower NIHSS scores, shorter OTR, absence of atrial fibrillation, and lower serum BNP levels were also significantly associated with favorable outcomes in AIS patients (all *p* < 0.01). Elevated serum BNP levels increased the risk of serious adverse events in both DT and BT groups (*p* < 0.001). ROC analysis showed that a combined model including NIHSS, BNP, collateral circulation, onset-to-reperfusion time, and atrial fibrillation achieved high predictive performance for good prognosis (AUC = 0.907, 95% CI: 0.881–0.934, *p* < 0.001).

**Conclusion:**

Collateral circulation is a strong predictor of both functional recovery and risk of adverse events in AIS patients undergoing thrombectomy, regardless of treatment strategy. While serum BNP may offer additional prognostic value, its moderate performance and overlap with cardiovascular conditions suggest that it should be interpreted cautiously. Pre-intervention collateral assessment remains a valuable tool for guiding individualized treatment decisions.

## 1 Introduction

Acute ischemic stroke (AIS) is a primary contributor to both mortality and long-term disability worldwide. Large vessel occlusions (LVO), particularly in the anterior circulation, carry a high risk of poor outcomes if not promptly treated (Hasan et al., 2018). Prompt and effective reperfusion is crucial to salvage ischemic penumbra and improve functional outcomes. Mechanical thrombectomy (MT) has become the standard of care for AIS with LVO, demonstrating significant benefits in recanalization and clinical recovery when performed within appropriate time windows (Vidale et al., 2020).

Two primary MT strategies have emerged: direct thrombectomy (DT), in which patients undergo MT without prior intravenous thrombolysis, and bridging thrombectomy (BT), which combines intravenous thrombolysis using recombinant tissue plasminogen activator (rt-PA) followed by MT. BT may be more beneficial for patients with milder stroke presentations or smaller infarct core volumes, improving 90-day functional outcomes and reducing mortality relative to DT (Sarraj et al., 2021). However, the optimal strategy remains uncertain, and results from recent trials have been inconsistent. Several randomized controlled trials have compared these two approaches. The DIRECT-MT trial conducted in China demonstrated that DT was non-inferior to BT with respect to 90-day functional independence, though BT achieved slightly higher early reperfusion rates (Yang et al., 2020). Similarly, the SKIP trial in Japan did not show non-inferiority of DT to BT, but the between-group differences were small and not statistically significant, raising questions about statistical power (Suzuki et al., 2021). The MR CLEAN-NO IV trial in the Netherlands found no significant differences in clinical outcomes between the two approaches, supporting the non-inferiority of DT under pragmatic clinical conditions (LeCouffe et al., 2021). The SWIFT-DIRECT trial also failed to demonstrate the non-inferiority of DT, although safety profiles and reperfusion rates were comparable (Mujanovic et al., 2024). Present meta-analyses also indicate no significant difference in clinical outcomes, suggesting that DT may be non-inferior to BT in select population (Chen et al., 2021; Podlasek et al., 2021). Collectively, these studies suggest that neither approach is universally superior, and that treatment decisions should be individualized based on patient-specific characteristics. Heterogeneity in study design, inclusion criteria, and rt-PA protocols complicates direct comparison and underscores the necessity for refined, individualized treatment selection. One critical factor that may influence outcomes is the status of collateral circulation.

Collateral circulation, a network of secondary blood vessels that helps sustain cerebral perfusion during vessel occlusion, plays a pivotal role in AIS outcomes by mitigating ischemic damage until reperfusion can be achieved (Bang et al., 2015). The status of collateral circulation is a critical determinant of infarct size, risk of hemorrhagic transformation, and overall functional prognosis. Patients with good collateral circulation often exhibit smaller infarct volumes and better clinical outcomes compared to those with poor collaterals. Studies have shown that robust collateral status is associated with higher rates of successful reperfusion, improved functional independence, and lower 90-days mortality rates, especially in patients treated with MT (Xu et al., 2022; Sallustio et al., 2017). However, despite strong supporting evidence, collateral assessment remains underutilized in real-world clinical decision-making (Uniken Venema et al., 2022). Several factors contribute to this gap, including the absence of standardized collateral grading methods, and limited consensus on how collateral status should influence treatment decisions (Sinha et al., 2022; Mangiardi et al., 2024). Multiphase computed tomographic angiography (mCTA) enables rapid, noninvasive assessment of collateral status at the bedside. The Menon collateral scale grades pial collateral filling from 0 to 5 based on the extent and timing of vessel opacification relative to the contralateral hemisphere, as assessed by mCTA. It is widely used in both clinical and research settings, with a score of 0–3 considered indicative of poor collateral circulation, while scores of 4 or higher denote good collateral status (Menon et al., 2015).

Serum brain natriuretic peptide (BNP) has been shown to be significantly elevated in patients with cardioembolic stroke, especially those with AF (Zhang et al., 2021). Higher plasma BNP levels on admission are independently predictive of a cardioembolic stroke mechanism and also serve as prognostic biomarkers for poor functional outcomes at 90 days, even after adjusting for confounding factors such as atrial fibrillation (AF) and congestive heart failure (Chaudhuri et al., 2015; Maruyama et al., 2017). BNP has shown additive value when integrated with clinical and imaging variables for stratifying ischemic stroke patients by risk and expected recovery trajectory (Rost et al., 2012; Nigro et al., 2014). However, its prognostic value in AIS patients with anterior circulation LVO remains unclear.

This retrospective comparative study aims to evaluate the prognostic impact of collateral circulation in AIS patients with anterior circulation LVO undergoing DT versus BT. By analyzing clinical outcomes in relation to collateral status assessed through mCTA, we seek to determine whether collateral circulation differentially affects the efficacy of DT and BT. The findings may provide valuable insights to guide therapeutic strategies and improve prognostic assessments in AIS management.

## 2 Methods

### 2.1 Study population

This retrospective cohort study analyzed data from electronic medical records of AIS patients treated at our hospital between March 2019 and March 2024. Eligible patients were aged > 18 years, diagnosed with acute anterior circulation LVO, and treated with either DT or BT. Only patients who underwent imaging upon admission, with collateral circulation assessments, and presented within 6 h of symptom onset were included. Exclusion criteria included insufficient imaging data for collateral assessment, contraindications to thrombectomy (e.g., major bleeding), significant comorbidities affecting prognosis (e.g., severe cardiac disease), or incomplete clinical records. Treatment selection was primarily based on predefined criteria: patients within 4.5 h of symptom onset who were eligible for intravenous thrombolysis received BT, whereas those beyond 4.5 h or with contraindications to thrombolysis received DT. Final treatment decisions were made by clinicians based on these criteria and individual patient factors. In the BT group, intravenous alteplase was administered at a standard dose of 0.9 mg/kg (maximum 90 mg). Per national stroke treatment guidelines, 10% of the total dose was administered as an intravenous bolus over 1 min, followed by continuous infusion of the remaining 90% over 60 min using an infusion pump. MT was initiated after thrombolysis.

This study was approved by the institutional review board of the First Hospital of Shanxi Medical University and conducted in accordance with the Declaration of Helsinki. Informed consent was waived due to the study’s retrospective, anonymized design.

### 2.2 Data collection

Data were systematically collected on demographic, clinical, and imaging factors. Demographic data included age, sex, smoking and drinking history, and comorbidities such as hypertension, diabetes, hyperlipidemia, coronary heart disease (CHD), previous stroke, and AF. Laboratory variables included fibrinogen, international normalized ratio (INR), serum BNP, HbA1c, creatinine (mg/dL), glucose, and platelet count. Additional clinical variables included systolic blood pressure (SBP), diastolic blood pressure (DBP), heart rate, Alberta Stroke Program Early CT Score (ASPECTS), and the time from last known well to CT scan.

Collateral circulation status was assessed using mCTA and graded according to the Menon collateral scale (range 0–5) (Menon et al., 2015). A score of 0 indicates the absence of visible vessels in any phase within the ischemic territory compared to the asymptomatic contralateral hemisphere, whereas a score of 5 represents normal or increased prominence and extent of pial vessels with no delay relative to the contralateral side. An mCTA score ≤3 was classified as poor collateral status, while a score ≥4 indicated good collateral status. Two experienced neuroradiologists independently reviewed the mCTA images while blinded to clinical and outcome data. Discrepancies were resolved by consensus.

Postoperative outcomes included NIHSS score, modified Rankin Scale (mRS) score, successful reperfusion (defined as mTICI 2b–3), onset-to-reperfusion time (OTR), rates of intracerebral hemorrhage (ICH) and hemorrhagic transformation, in-hospital mortality, length of stay, and adverse events [reported according to Common Terminology Criteria for Adverse Events (CTCAE) version 5.0 criteria]. All adverse events are listed in Supplementary Table S1.

Functional prognosis was assessed at 90 days using the mRS during in-person outpatient follow-up. A good outcome was defined as an mRS score of 0 to 2, while a poor outcome was defined as an mRS score > 2.

### 2.3 Statistical analysis

All statistical analyses were performed using SPSS software (version 25.0; IBM Corp., Armonk, NY, United States). Categorical variables were presented as frequencies (percentages) and compared between groups using the chi-square test or Fisher’s exact test. The normality of continuous variables was assessed using the Kolmogorov–Smirnov test. Normally distributed variables were analyzed using the independent-samples t-test, while non-normally distributed variables were compared using the Mann–Whitney U test. Logistic regression models were applied to identify risk factors influencing patient prognosis (mRS ≤ 2) and the occurrence of adverse events (CTCAE > 2) under different treatment strategies. Variables with a *p* value < 0.05 in the univariate analysis were entered into the multivariate logistic regression model. Collinearity was evaluated by calculating variance-inflation factors for every candidate variable. To evaluate the predictive value of significant factors on good prognosis (mRS ≤ 2), receiver operating characteristic (ROC) curves were generated, and the area under the curve (AUC) was calculated with 95% confidence intervals (CIs). Optimal cutoff values were determined using the Youden Index. A two-sided p value < 0.05 was considered statistically significant.

## 3 Results

### 3.1 Patient demographics and baseline characteristics

Between March 2019 and March 2024, a total of 460 patients with AIS were included in the study. Based on the mRS scores, patients were classified into those with a good outcome (mRS ≤ 2, *n* = 164) and a poor outcome (mRS > 2, *n* = 296) (Supplementary Figure S1). Baseline characteristics of patients with good and poor outcomes are presented in Table 1. Patients with good outcomes had a significantly lower baseline NIHSS score (*p* < 0.001) and higher ASPECTS scores (*p* = 0.005). Good collateral circulation was more prevalent in the good outcome group, while the incidence of AF was significantly lower (*p* < 0.001). Furthermore, patients with good outcomes exhibited lower international normalized ratio (*p* = 0.039) and had lower levels of serum BNP (*p* < 0.001), heart rates (*p* = 0.007), and baseline glucose levels (*p* < 0.001).

**Table 1:** Baseline characteristics of patients with different prognostic outcomes.

Characteristics	Good outcome (mRS ≤ 2) (*n* = 164)	Poor outcome (mRS > 2) (*n* = 296)	*P*
Demographics			
Age, year	65.00 ± 0.74	64.27 ± 0.57	0.436
Male	88 (53.7%)	151 (51.0%)	0.627
Smoking	101 (61.6%)	168 (56.8%)	0.325
Drinking	117 (71.3%)	206 (69.6%)	0.750
Comorbidities			
Hypertension	101 (61.6%)	168 (56.8%)	0.325
Diabetes	108 (65.9%)	207 (69.9%)	0.402
Hyperlipidemia	91 (55.5%)	173 (58.4%)	0.556
CHD	123 (76.8%)	234 (79.1%)	0.637
Previous stroke	32 (19.5%)	75 (25.3%)	0.168
AF	35 (21.3%)	116 (39.2%)	<0.001
Clinical data			
Baseline NIHSS score	8.50 (7.00, 10.00)	17.00 (11.00, 21.00)	<0.001
Time from last known well to CT scan, minutes	190.00 (111.75, 287.50)	188.00 (115.00, 274.25)	0.880
SBP, mmHg	136.88 ± 1.57	137.79 ± 1.17	0.644
DBP, mmHg	84.22 ± 1.22	82.97 ± 0.95	0.420
Heart rate, bpm	91.00 (69.75, 102.00)	94.00 (77.00, 104.00)	0.007
Classification			0.771
Direct embolectomy	80 (48.8%)	140 (47.3%)	
Bridge embolectomy	84 (51.2%)	156 (52.7%)	
Imaging			
ASPECTS	8.00 (7.00, 9.00)	7.00 (6.00, 9.00)	0.005
mCTA score			<0.001
1	8 (4.9%)	91 (30.7%)	
2	32 (19.5%)	75 (25.3%)	
3	37 (22.6%)	74 (25.0%)	
4	58 (35.4%)	35 (11.8%)	
5	29 (17.7%)	21 (7.1%)	
Good collateral circulation	87 (53.0%)	56 (18.9%)	<0.001
Laboratory			
Fibrinogen, g/L	3.54 ± 0.06	3.50 ± 0.05	0.650
INR	1.04 ± 0.02	1.10 ± 0.02	0.039
Serum BNP, pg/mL	111.76 (85.20, 149.14)	171.95 (115.59, 356.04)	<0.001
HbA1c, %	6.45 ± 0.12	6.43 ± 0.09	0.891
Creatinine, mg/dL	1.01 ± 0.02	1.01 ± 0.02	0.992
Baseline glucose, mg/dL	129.31 ± 2.22	141.23 ± 1.58	<0.001
Platelet count, ×10^9^/L	295.00 (226.75, 369.00)	305.00 (223.50, 381.50)	0.575

Continuous variables are presented as mean ± standard deviation, non-normally distributed variables are presented as median (interquartile range, IQR), Categorical variables are presented as n (%). AF, Atrial fibrillation; ASPECTS, Alberta Stroke Program Early CT Score; BNP, B-type brain natriuretic peptide; CTA, Computed tomography angiography; CHD, Coronary heart disease; HbA1c, Hemoglobin A1c; INR, International Normalized Ratio; NIHSS, National Institute of Health Stroke Scale.

Among the 460 patients, 220 underwent DT, while 240 received BT. No significant differences were observed between the two groups in terms of baseline characteristics (Table 2). Postoperative outcomes, including mRS scores, successful reperfusion rates, OTR, in-hospital mortality rates, postoperative NIHSS scores, length of hospital stay, and adverse event risk levels, were similar between the two groups. However, the BT group showed significantly higher rates of ICH (*p* < 0.001) and hemorrhagic transformation (*p* = 0.011).

**Table 2:** Baseline characteristics and postoperative outcomes between patients undergoing direct thrombectomy and bridging thrombectomy.

Characteristics	All patients (*n* = 460)	Direct thrombectomy (*n* = 220)	Bridging thrombectomy (*n* = 240)	P	SMD
Demographics					
Age, year	64.53 ± 9.70	64.88 ± 0.65	64.21 ± 0.63	0.463	1.047
Male	239 (52.0%)	112 (50.9%)	127 (52.9%)	0.709	−0.040
Smoking	269 (58.5%)	128 (58.2%)	141 (58.8%)	0.925	−0.012
Drinking	323 (70.2%)	158 (71.8%)	165 (68.8%)	0.472	0.067
Comorbidities					
Hypertension	269 (58.5%)	123 (55.9%)	146 (60.8%)	0.299	−0.100
Diabetes	315 (68.5%)	151 (68.6%)	164 (68.3%)	1.000	0.007
Hyperlipidemia	264 (57.4%)	117 (53.2%)	147 (61.3%)	0.090	−0.163
CHD	360 (78.3%)	175 (79.5%)	185 (77.1%)	0.572	0.060
Previous stroke	107 (23.3%)	46 (20.9%)	61 (25.4%)	0.271	−0.107
AF	151 (32.8%)	63 (28.6%)	88 (36.7%)	0.074	−0.171
Clinical data					
Baseline NIHSS score	11.00 (8.00, 19.00)	11.00 (9.00, 19.00)	11.00 (8.00, 19.00)	0.797	0.026
Time from last known well to CT scan, minutes	189.00 (112.00, 277.75)	184.50 (111.75, 288.25)	191.50 (115.00, 268.00)	0.602	0.055
SBP, mmHg	137.47 ± 20.09	136.10 ± 1.37	138.72 ± 1.28	0.164	−1.976
DBP, mmHg	84.00 (73.00, 94.00)	84.00 (74.00, 94.26)	83.50 (73.00, 93.00)	0.531	0.047
Heart rate, bpm	93.00 (75.00, 104.00)	93.50 (74.75, 101.25)	93.00 (75.00, 106.25)	0.537	−0.049
Imaging					
ASPECTS	7.00 (6.00, 9.00)	7.00 (7.00, 9.00)	7.00 (6.00, 9.00)	0.252	0.105
Good collateral circulation	143 (31.1%)	67 (30.5%)	76 (31.7%)	0.840	−0.026
Laboratory					
Fibrinogen, g/L	3.51 ± 0.84	3.58 ± 0.06	3.45 ± 0.05	0.118	2.354
INR	1.08 ± 0.30	1.09 ± 0.02	1.06 ± 0.02	0.260	1.500
Serum BNP, pg/mL	115.19 (103.94, 289.07)	116.14 (85.20, 174.69)	111.76 (89.42, 409.65)	0.200	−0.146
HbA1c, %	6.44 ± 1.54	6.43 ± 0.10	6.44 ± 0.11	0.941	−0.095
Creatinine, mg/dL	1.01 ± 0.30	1.01 ± 0.02	1.00 ± 0.02	0.689	0.500
Baseline glucose, mg/dL	136.98 ± 28.17	136.16 ± 1.88	137.73 ± 1.84	0.553	−0.844
Platelet count, ×10^9^/L	301.00 (224.50, 378.75)	291.00 (211.00, 366.75)	303.50 (234.75, 386.25)	0.111	−0.153
Postoperative outcomes					
Postoperative NIHSS score	10.00 (6.00, 15.00)	10.00 (6.00, 13.00)	10.00 (6.00, 16.25)	0.432	−0.090
mRS score	3.00 (2.00, 5.00)	3.00 (2.00, 5.00)	3.00 (2.00, 5.00)	0.654	−0.042
Successful reperfusion rate	317 (68.9%)	153 (69.5%)	164 (68.3%)	0.840	0.026
OTR, minutes	184.50 (161.00, 199.00)	185.00 (165.00, 201.25)	183.50 (163.00, 202.25)	0.368	0.010
ICH rate	233 (50.7%)	73 (33.2%)	160 (66.7%)	<0.001	−0.670
Hemorrhagic transformation rate	353 (76.7%)	157 (71.4%)	196 (81.7%)	0.011	−0.243
In-hospital mortality rate	125 (27.2%)	54 (24.5%)	71 (29.6%)	0.249	−0.113
LOS, day	12.00 (8.00, 16.00)	12.00 (8.00, 16.00)	12.00 (8.75, 16.00)	0.842	−0.018
Adverse event risk level				0.479	−0.080
1	155 (33.7%)	77 (35.0%)	78 (32.5%)		
2	99 (21.5%)	45 (20.5%)	54 (22.5%)		
3	54 (6.7%)	31 (14.1%)	23 (9.6%)		
4	27 (5.2%)	13 (5.9%)	14 (5.8%)		
5	125 (27.2%)	54 (24.5%)	71 (29.6%)		

Continuous variables are presented as mean ± standard deviation, non-normally distributed variables are presented as median (interquartile range, IQR), Categorical variables are presented as n (%). AF, Atrial fibrillation; ASPECTS, Alberta Stroke Program Early CT Score; BNP, B-type brain natriuretic peptide; CTA, Computed tomography angiography; CHD, Coronary heart disease; HbA1c, Hemoglobin A1c; INR, International Normalized Ratio; NIHSS, National Institute of Health Stroke Scale; OTR, Onset-to-Reperfusion Time; ICH, Intracranial Hemorrhage; LOS, Length of Hospital Stay. Direct Mechanical Thrombectomy and Bridging thrombectomy were statistically compared.

### 3.2 Association between collateral circulation and functional prognosis

**Figure 1:**
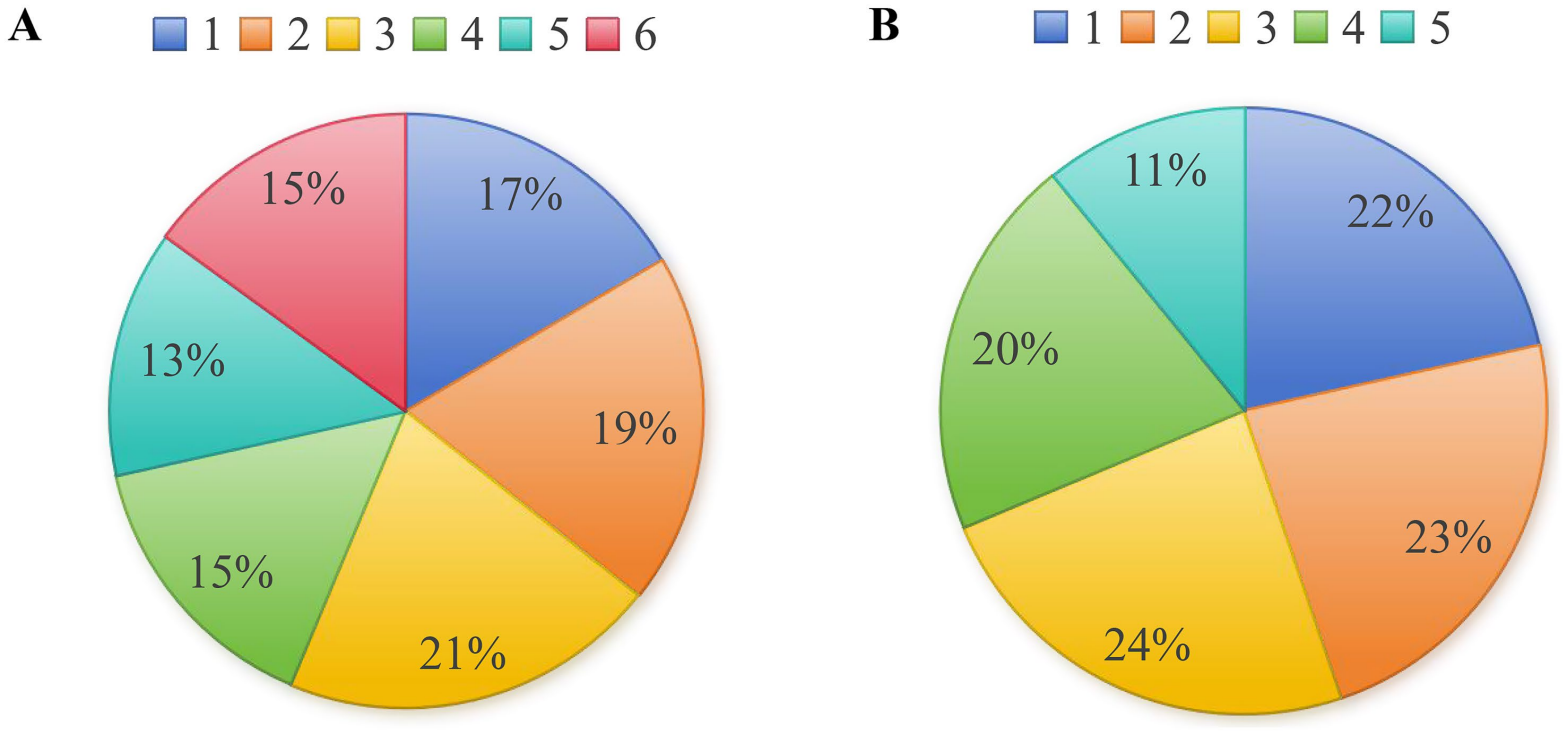
Distribution of mRS scores and mCTA scores in patients. **(A)** Distribution of mRS scores; **(B)** Distribution of collateral grading.

The distributions of mRS scores and mCTA grades among all patients are shown in Figure 1. Regarding functional prognosis, mRS scores varied, with 17% of patients achieving a score of 1 and 19% with a score of 2 (Figure 1A). Collateral grading (mCTA) was variable, with scores distributed across all levels, indicating heterogeneity in collateral status within the cohort (Figure 1B). Correlation analysis demonstrated a significant inverse relationship between collateral circulation (mCTA score) and functional outcome (mRS score). Higher mCTA scores, indicating better collateral circulation, were associated with lower mRS scores, reflecting better functional outcomes (Spearman’s R = −0.299, *p* < 0.01). This inverse correlation remained significant in both DT group (Spearman’s R = −0.363, *p* < 0.01) and BT group (Spearman’s R = −0.238, *p* < 0.01).

### 3.3 Factors influencing prognosis under different treatment strategies

Logistic regression analyses were conducted to identify factors associated with favorable prognosis (mRS ≤ 2) in all patients, as well as within the DT and BT subgroups (Table 3). In the overall cohort (n = 460), baseline NIHSS score was negatively associated with better outcomes (OR: 0.740, 95%CI: 0.694–0.789, *p* < 0.001), and good collateral circulation had a strong positive effect on prognosis (OR: 3.987, 95%CI: 2.262–7.029, *p* < 0.001). Absence of AF (OR: 0.400, 95%CI: 0.218–0.736, *p* = 0.003) and lower serum BNP levels (OR: 0.994, 95%CI: 0.992–0.996, *p* < 0.001) were also a predictor of good outcomes. Additionally, a shorter OTR was beneficial (OR: 0.987, 95%CI: 0.978–0.996, *p* = 0.006). An interaction term between treatment strategy (DT vs. BT) and collateral status was then included in the multivariate model to assess potential effect modification. The interaction was not statistically significant (*p* = 0.738), indicating that the effect of collateral status on prognosis did not differ significantly between the two treatment groups. Based on this result, subgroup analyses stratified by treatment strategy were conducted to further explore prognostic factors within each group. In the DT group (*n* = 220), a lower baseline NIHSS score (OR: 0.785, 95%CI: 0.729–0.846, *p* < 0.001) and good collateral circulation (OR: 4.717, 95%CI: 2.259–9.850, *p* < 0.001) were independently associated with a higher likelihood of favorable functional outcome. In the BT group (*n* = 240), lower baseline NIHSS score (OR: 0.688, 95%CI: 0.617–0.766, *p* < 0.001), good collateral circulation (OR: 4.668, 95%CI: 2.029–10.741, *p* < 0.001), shorter OTR (OR: 0.985, 95% CI: 0.971–0.999, *p* = 0.031) and the absence of AF (OR: 0.195, 95% CI: 0.081–0.471, *p* < 0.001) were significantly associated with improved outcomes.

**Table 3:** Logistic regression analysis of factors affecting patient prognosis under different treatment strategies.

Variables	Univariate logistic		Multivariate logistic	
	OR (95%CI)	*p*	OR (95%CI)	*p*
All patients (*n* = 460)				
Age, year	1.009 (0.983–1.035)	0.520		
Male	1.142 (0.679–1.921)	0.618		
Baseline NIHSS	0.738 (0.696–0.781)	<0.001	0.740 (0.694–0.789)	<0.001
ASPECTS	1.183 (0.988–1.417)	0.068		
Smoking	1.075 (0.630–1.836)	0.791		
Drinking	1.393 (0.782–2.479)	0.260		
SBP, mmHg	0.997 (0.984–1.010)	0.669		
DBP, mmHg	1.008 (0.992–1.024)	0.326		
Good collateral circulation	5.213 (3.014–9.016)	<0.001	3.987 (2.262–7.029)	<0.001
Time from last known well to CT scan, minutes	1.000 (0.997–1.003)	0.907		
Hypertension	1.260 (0.742–2.139)	0.392		
Diabetes	0.736 (0.422–1.286)	0.282		
Hyperlipidemia	1.135 (0.679–1.898)	0.628		
CHD	0.898 (0.483–1.671)	0.735		
Previous stroke	0.865 (0.466–1.604)	0.646		
AF	0.381 (0.215–0.675)	0.001	0.400 (0.218–0.736)	0.003
Fibrinogen	0.999 (0.727–1.372)	0.996		
INR	0.652 (0.283–1.501)	0.315		
Serum BNP, pg/mL	0.993 (0.991–0.995)	<0.001	0.994 (0.992–0.996)	<0.001
Heart rate, bpm	0.964 (0.944–0.985)	0.001		
HbA1c, %	1.038 (0.876–1.230)	0.667		
Creatinine, mg/dL	1.335 (0.565–3.155)	0.511		
Baseline glucose, mg/dL	0.964 (0.951–0.977)	<0.001		
Platelet count, ×10^9^/L	0.999 (0.996–1.002)	0.447		
OTR, minutes	0.985 (0.976–0.994)	0.002	0.987 (0.978–0.996)	0.006
Direct thrombectomy (*n* = 220)				
Age, year	0.989 (0.952–1.026)	0.544		
Male	1.390 (0.662–2.917)	0.384		
Baseline NIHSS	0.771 (0.717–0.830)	<0.001	0.785 (0.729–0.846)	<0.001
ASPECTS	1.161 (0.898–1.503)	0.255		
Smoking	0.767 (0.361–1.627)	0.489		
Drinking	1.302 (0.589–2.881)	0.514		
SBP, mmHg	1.005 (0.988–1.023)	0.564		
DBP, mmHg	1.010 (0.990–1.031)	0.343		
Good collateral circulation	6.804 (3.048–15.188)	<0.001	4.717 (2.259–9.850)	<0.001
Time from last known well to CT scan, minutes	1.001 (0.997–1.005)	0.615		
Hypertension	1.513 (0.730–3.137)	0.266		
Diabetes	0.541 (0.240–1.222)	0.140		
Hyperlipidemia	1.718 (0.825–3.575)	0.148		
CHD	0.880 (0.358–2.161)	0.780		
Previous stroke	0.921 (0.387–2.190)	0.851		
AF	0.627 (0.273–1.439)	0.270		
Fibrinogen	1.041 (0.675–1.605)	0.857		
INR	1.221 (0.427–3.490)	0.709		
Serum BNP, pg/mL	1.002 (1.000–1.003)	0.122		
Heart rate, bpm	0.940 (0.911–0.971)	<0.001		
HbA1c, %	0.938 (0.734–1.198)	0.608		
Creatinine, mg/dL	0.661 (0.210–2.087)	0.481		
Baseline glucose, mg/dL	0.984 (0.967–1.002)	0.074		
Platelet count, ×10^9^/L	0.999 (0.995–1.003)	0.715		
OTR, minutes	0.990 (0.978–1.003)	0.131		
Bridging thrombectomy (*n* = 240)				
Age, year	1.031 (0.990–1.074)	0.143		
Male	0.827 (0.388–1.766)	0.624		
Baseline NIHSS	0.698 (0.635–0.766)	<0.001	0.688 (0.617–0.766)	<0.001
ASPECTS	1.267 (0.979–1.641)	0.073		
Smoking	0.796 (0.364–1.740)	0.568		
Drinking	2.860 (1.143–7.157)	0.025		
SBP, mmHg	0.991 (0.973–1.010)	0.346		
DBP, mmHg	1.011 (0.984–1.039)	0.445		
Good collateral circulation	7.601 (3.259–17.728)	<0.001	4.668 (2.029–10.741)	<0.001
Time from last known well to CT scan, minutes	0.998 (0.994–1.003)	0.483		
Hypertension	1.438 (0.646–3.202)	0.374		
Diabetes	1.307 (0.573–2.980)	0.525		
Hyperlipidemia	0.787 (0.366–1.693)	0.539		
CHD	0.841 (0.353–2.003)	0.696		
Previous stroke	0.701 (0.294–1.673)	0.423		
AF	0.192 (0.081–0.453)	<0.001	0.195 (0.081–0.471)	<0.001
Fibrinogen	0.933 (0.580–1.499)	0.774		
INR	0.331 (0.085–1.287)	0.110		
Serum BNP, pg/mL	1.000 (0.998–1.002)	0.807		
Heart rate, bpm	0.976 (0.946–1.007)	0.131		
HbA1c, %	1.028 (0.810–1.304)	0.823		
Creatinine, mg/dL	2.726 (0.672–11.056)	0.160		
Baseline glucose, mg/dL	0.951 (0.930–0.972)	<0.001		
Platelet count, ×10^9^/L	1.001 (0.997–1.006)	0.545		
OTR, minutes	0.973 (0.958–0.987)	<0.001	0.985 (0.971–0.999)	0.031

AF, Atrial fibrillation; ASPECTS, Alberta Stroke Program Early CT Score; BNP, B-type brain natriuretic peptide; CTA, computed tomography angiography; CHD, Coronary heart disease; HbA1c, hemoglobin A1c; INR, International Normalized Ratio; NIHSS, National Institute of Health Stroke Scale; OTR, Onset-to-Reperfusion Time. Factors with a *p*-value <0.05 in the univariate logistic regression were included in the multivariate logistic regression analysis for all patients, direct thrombectomy, and bridging thrombectomy group.

### 3.4 Factors influencing the occurrence of adverse events under different treatment strategies

To identify predictors of serious adverse events (defined as CTCAE grade > 2), logistic regression analyses were performed for the entire cohort and separately within the DT and BT subgroups (Table 4). In the overall cohort (*n* = 460), mRS ≤ 2 were associated with a decreased risk of serious adverse events (OR: 0.634, 95%CI: 0.538–0.746, *p* < 0.001), good collateral status was a protective factor against adverse events (OR = 0.393, 95% CI: 0.234–0.661, *p* < 0.001). Conversely, elevated serum BNP levels were associated with an increased risk of serious adverse events (OR = 1.012, 95% CI: 1.010–1.015, *p* < 0.001). Similarly, in the DT group (*n* = 220), higher serum BNP levels were associated with an increased risk of serious adverse events (OR = 1.020, 95% CI: 1.012–1.028, *p* < 0.001). In the BT group (*n* = 240), higher serum BNP levels were also linked with a higher risk of serious adverse events (OR: 1.018, 95% CI: 1.011–1.024, *p* < 0.001).

**Table 4:** Logistic regression analysis of influencing factors of adverse events (CTCAE>2) in patients under different treatment strategies.

Variables	Univariate logistic		Multivariate logistic	
	OR (95%CI)	*p*	OR (95%CI)	*p*
All patients (*n* = 460)				
Age, year	1.004 (0.959–1.089)	0.206		
Male	1.069 (0.662–1.727)	0.785		
mRS≤2	0.679 (0.568–0.813)	<0.001	0.634 (0.538–0.746)	<0.001
Baseline NIHSS	1.017 (0.956–1.098)	0.412		
ASPECTS	1.098 (0.925–1.304)	0.285		
Smoking, mmHg	1.236 (0.755–2.023)	0.400		
Drinking, mmHg	0.941 (0.553–1.602)	0.823		
SBP	1.007 (0.995–1.019)	0.239		
DBP	0.982 (0.968–0.996)	0.012		
Good collateral circulation	0.384 (0.223–0.660)	0.001	0.393 (0.234–0.661)	<0.001
Time from last known well to CT scan, minutes	1.001 (0.998–1.003)	0.726		
Hypertension	1.091 (0.668–1.781)	0.729		
Diabetes	1.256 (0.734–2.149)	0.406		
Hyperlipidemia	0.873 (0.541–1.408)	0.577		
CHD	0.745 (0.421–1.320)	0.314		
Previous stroke	0.871 (0.485–1.565)	0.644		
AF	1.039 (0.617–1.751)	0.885		
Fibrinogen	1.012 (0.755–1.357)	0.935		
INR	1.083 (0.493–2.376)	0.843		
Serum BNP, pg/mL	1.013 (1.010–1.015)	<0.001	1.012 (1.010–1.015)	<0.001
Heart rate, bpm	0.984 (0.966–1.003)	0.094		
HbA1c, %	1.051 (0.897–1.232)	0.536		
Creatinine, mg/dL	1.272 (0.573–2.826)	0.555		
Baseline glucose, mg/dL	0.998 (0.988–1.009)	0.737		
Platelet count, ×10^9^/L	1.001 (0.998–1.003)	0.610		
OTR, minutes	0.998 (0.990–1.007)	0.706		
Direct thrombectomy (*n* = 220)				
Age, year	1.025 (0.875–1.079)	0.007		
Male	1.079 (0.383–3.036)	0.886		
mRS≤2	0.801 (0.554–1.158)	0.238		
Baseline NIHSS	1.037 (0.942–1.085)	0.594		
ASPECTS	1.555 (1.010–2.392)	0.045		
Smoking, mmHg	0.626 (0.208–1.884)	0.405		
Drinking, mmHg	1.613 (0.485–5.362)	0.435		
SBP	0.985 (0.959–1.012)	0.283		
DBP	0.987 (0.956–1.020)	0.449		
Good collateral circulation	0.654 (0.193–2.212)	0.495		
Time from last known well to CT scan, minutes	1.005 (1.000–1.010)	0.062		
Hypertension	0.986 (0.368–2.640)	0.978		
Diabetes	0.632 (0.184–2.169)	0.466		
Hyperlipidemia	1.275 (0.453–3.589)	0.645		
CHD	0.966 (0.275–3.398)	0.958		
Previous stroke	0.806 (0.251–2.585)	0.717		
AF	3.037 (0.995–9.276)	0.051		
Fibrinogen	1.228 (0.662–2.280)	0.515		
INR	0.522 (0.105–2.607)	0.428		
Serum BNP, pg/mL	1.024 (1.015–1.034)	<0.001	1.020 (1.012–1.028)	<0.001
Heart rate, bpm	1.025 (0.982–1.070)	0.256		
HbA1c, %	1.261 (0.897–1.774)	0.182		
Creatinine, mg/dL	0.919 (0.178–4.738)	0.920		
Baseline glucose, mg/dL	0.977 (0.953–1.003)	0.078		
Platelet count, ×10^9^/L	1.004 (0.998–1.009)	0.234		
OTR, minutes	0.994 (0.976–1.012)	0.498		
Bridging thrombectomy (*n* = 240)				
Age, year	1.015 (0.914–1.068)	0.194		
Male	0.611 (0.225–1.662)	0.335		
mRS≤2	0.809 (0.576–1.137)	0.222		
Baseline NIHSS	1.050 (0.908–1.094)	0.027		
ASPECTS	0.956 (0.693–1.319)	0.784		
Smoking, mmHg	1.621 (0.574–4.578)	0.362		
Drinking, mmHg	0.796 (0.277–2.288)	0.672		
SBP	1.018 (0.992–1.044)	0.176		
DBP	0.994 (0.963–1.026)	0.719		
Good collateral circulation	1.225 (0.429–3.496)	0.705		
Time from last known well to CT scan, minutes	0.997 (0.991–1.002)	0.193		
Hypertension	2.008 (0.714–5.651)	0.187		
Diabetes	0.530 (0.181–1.554)	0.247		
Hyperlipidemia	1.143 (0.428–3.052)	0.789		
CHD	0.439 (0.149–1.291)	0.135		
Previous stroke	0.685 (0.225–2.088)	0.506		
AF	1.813 (0.666–4.934)	0.244		
Fibrinogen	1.068 (0.618–1.844)	0.814		
INR	3.985 (0.775–20.483)	0.098		
Serum BNP, pg/mL	1.020 (1.013–1.027)	<0.001	1.018 (1.011–1.024)	<0.001
Heart rate, bpm	0.979 (0.943–1.015)	0.251		
HbA1c, %	1.002 (0.730–1.374)	0.991		
Creatinine, mg/dL	0.398 (0.071–2.245)	0.297		
Baseline glucose, mg/dL	1.000 (0.981–1.019)	0.964		
Platelet count, ×10^9^/L	0.998 (0.993–1.004)	0.568		
OTR, minutes	0.995 (0.978–1.013)	0.600		

AF, Atrial fibrillation; ASPECTS, Alberta Stroke Program Early CT Score; BNP, B-type brain natriuretic peptide; CHD, Coronary heart disease; HbA1c, hemoglobin A1c; INR, International Normalized Ratio; NIHSS, National Institute of Health Stroke Scale; OTR, Onset-to-Reperfusion Time. Factors with a *p*-value <0.05 in the univariate logistic regression were included in the multivariate logistic regression analysis for all patients and direct thrombectomy, while Factors with a *p*-value <0.1 in the univariate logistic regression were included in the multivariate logistic regression analysis for bridging thrombectomy group.

### 3.5 Diagnostic performance of prognostic factors

**Figure 2:**
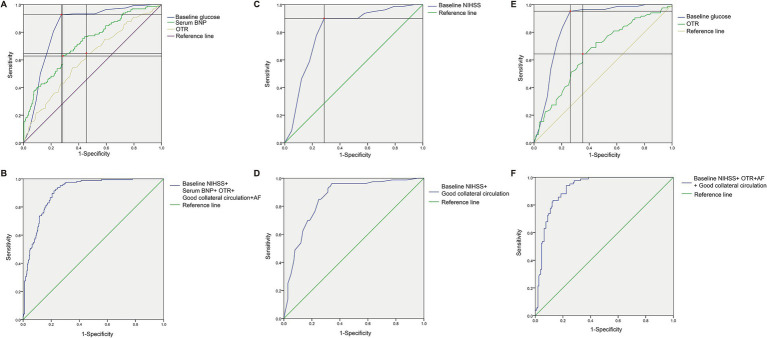
ROC curves of predictors for favorable functional outcome (mRS ≤2). **(A)** Variables with diagnostic value in all patients; **(B)** Combined ROC curve of independent predictors in all patients; **(C)** Variables with diagnostic value in patients undergoing direct thrombectomy; **(D)** Combined ROC curve of independent predictors in patients undergoing direct thrombectomy; **(E)** Variables with diagnostic value in patients undergoing bridging therapy; **(F)** Combined ROC curve of independent predictors in patients undergoing bridging therapy.

To further evaluate the diagnostic performance of the above prognostic factors associated with patient good outcomes (mRS ≤ 2), ROC curve analysis was performed (Table 5 and Figure 2). In the overall cohort (*n* = 460), baseline NIHSS score showed the highest predictive accuracy, with an AUC of 0.835 (95% CI: 0.797–0.873, *p* < 0.001), a sensitivity of 92.68%, and a specificity of 72.64% at the optimal cut-off value of 13.0. Serum BNP showed moderate discriminatory power (AUC = 0.723, 95% CI: 0.674–0.771, *p* < 0.001), while OTR demonstrated limited predictive performance (AUC = 0.613, 95% CI: 0.559–0.666, p < 0.001). A combined model incorporating baseline NIHSS, serum BNP, OTR, collateral status, and AF significantly improved overall classification performance, achieving an AUC of 0.907 (95% CI: 0.881–0.934, *p* < 0.001), with a sensitivity of 91.46% and specificity of 78.04% (Figures 2A,B). In the DT group (*n* = 220), baseline NIHSS achieved an AUC of 0.809 (95% CI: 0.750–0.869, *p* < 0.001). Combining it with collateral status yielded a modest improvement in predictive performance, with an AUC of 0.857 (95% CI: 0.806–0.907, *p* < 0.001), a sensitivity of 96.25%, and a specificity of 65.71% (Figures 2C,D). In the BT group (*n* = 240), baseline NIHSS also demonstrated strong predictive value, with an AUC of 0.859 (95% CI: 0.811–0.907, *p* < 0.001). OTR alone exhibited limited discriminative ability (AUC = 0.660, 95% CI: 0.588–0.732, *p* < 0.001). A multivariable model combining NIHSS, OTR, AF, and collateral status achieved an AUC of 0.918 (95% CI: 0.884–0.952, *p* < 0.001), with a sensitivity of 94.05% and a specificity of 77.56% (Figures 2E,F).

**Table 5:** ROC evaluation of diagnostic performance of different factors.

Variables	AUC	95%CI	Cut-off	Sensitivity	Specificity	*p*
All patients (*n* = 460)						
Baseline NIHSS	0.835	0.797–0.873	13.00	92.68%	72.64%	<0.001
Serum BNP, pg/mL	0.723	0.674–0.771	122.79	62.80%	71.62%	<0.001
OTR, minutes	0.613	0.559–0.666	182.50	64.63%	54.39%	<0.001
Baseline NIHSS+ serum BNP + OTR + good collateral circulation + AF	0.907	0.881–0.934	0.304	91.46%	78.04%	<0.001
Direct thrombectomy (*n* = 220)						
Baseline NIHSS	0.809	0.750–0.869	13.00	90.00%	71.43%	<0.001
Baseline NIHSS + good collateral circulation	0.857	0.806–0.907	-	96.25%	65.71%	<0.001
Bridging thrombectomy (*n* = 240)						
Baseline NIHSS	0.859	0.811–0.907	13.00	95.24%	73.72%	<0.001
OTR, minutes	0.660	0.588–0.732	175.50	64.29%	64.74%	<0.001
Baseline NIHSS + OTR + AF + good collateral circulation	0.918	0.884–0.952	-	94.05%	77.56%	<0.001

BNP, B-type brain natriuretic peptide; NIHSS, National Institute of Health Stroke Scale; OTR, Onset-to-Reperfusion Time; ROC, Receiver Operating Characteristic.

## 4 Discussion

In this retrospective comparative study of 460 patients with AIS due to anterior circulation LVO, we evaluated the prognostic significance of collateral circulation in patients treated with DT or BT. Our findings highlight the critical role of collateral status in determining clinical outcomes, regardless of the thrombectomy strategy used.

Comparison between DT and BT revealed no significant differences in baseline characteristics or most postoperative outcomes, including mRS scores, successful reperfusion rates, OTR, in-hospital mortality, postoperative NIHSS scores, length of stay, or adverse event rates. However, the BT group had a higher incidence of ICH and hemorrhagic transformation, consistent with previous studies reporting an elevated risk of hemorrhagic complications when intravenous thrombolysis precedes MT (Podlasek et al., 2021; Zhang et al., 2022). Although this difference in hemorrhage rates may seem inconsistent with the comparable 90-day mRS outcomes between groups, it is likely that most hemorrhagic events in the BT group were asymptomatic petechial hemorrhages or small parenchymal hematomas that did not cause significant neurological deterioration. Such hemorrhages are less likely to impact long-term functional status. Because of the retrospective study design and incomplete documentation of neurological status at the time of hemorrhage, we could not reliably distinguish symptomatic from asymptomatic ICH. This limitation underscores the need for future prospective studies incorporating standardized assessments of symptomatic ICH.

Logistic regression analysis identified several key prognostic factors associated with functional outcomes. In both the overall cohort and the BT and DT subgroups, baseline NIHSS score and good collateral status emerged as strong predictors of favorable prognosis. This underscores the importance of initial stroke severity and collateral status in patient recovery. These findings are consistent with previous studies showing that lower baseline NIHSS scores, reflecting milder initial neurological deficits, are associated with better functional outcomes (Yeo et al., 2015). Good collateral status was consistently associated with better outcomes, as adequate collateral flow helps preserve cerebral perfusion in ischemic regions, thereby limiting infarct expansion and improving the likelihood of successful reperfusion. Correlation analysis further confirmed that good collateral status is strongly associated with favorable 90-day functional outcomes. This is consistent with previous studies reporting that patients with robust collateral circulation have significantly higher odds of successful reperfusion and reduced 90-day mortality (Xu et al., 2022; Yeo et al., 2015). Collateral circulation provides critical support in maintaining cerebral perfusion, which limits infarct expansion and enhances reperfusion success, factors that have been widely observed to improve clinical outcomes (Qian et al., 2020). This inverse relationship between collateral quality and mRS scores was observed across the entire cohort and within both DT and BT subgroups, underscoring the universal importance of collateral circulation in AIS management. Furthermore, logistic regression revealed that good collateral status and lower mRS scores serve as protective factors against serious adverse events. A low mRS score, indicative of favorable prognosis, may be associated with a lower risk of adverse events, potentially due to the protective role of collateral circulation in maintaining hemodynamic stability and reducing ischemic injury (Yeo et al., 2015). This ability to limit infarct progression and maintain hemodynamic stability suggests that collateral status may inform treatment selection, particularly for patients at elevated risk of ICH. Patients with poor collateral status are more susceptible to reperfusion injury and hemorrhagic transformation, particularly following intravenous thrombolysis, and may therefore derive greater benefit from a direct thrombectomy strategy. Conversely, patients with good collateral status may be suitable candidates for bridging therapy and could potentially benefit from enhanced microvascular perfusion, supporting the role of pre-intervention collateral assessment in guiding personalized therapeutic strategies in AIS.

Additionally, lower serum BNP levels, absence of AF, and shorter OTR were each independently associated with favorable outcomes in the overall cohort. These findings are consistent with existing literature indicating that elevated BNP levels are associated with poorer prognoses in stroke patients. Elevated BNP, often a marker of cardiac dysfunction and increased hemodynamic stress, has been shown to correlate with greater stroke severity and worse clinical outcomes (Maruyama et al., 2017; Shibazaki et al., 2012). Similarly, our study found that elevated serum BNP levels were associated with an increased risk of adverse events, potentially reflecting a greater burden of comorbidities, which may increase susceptibility to complications, as previously reported (Xu et al., 2020). Moreover, AF is known to increase the risk of hemorrhagic complications following intravenous thrombolysis, without consistently improving long-term functional outcomes (Akbik et al., 2022). Additionally, a shorter OTR was significantly associated with improved prognosis, likely due to a higher likelihood of successful reperfusion and reduced mortality risk. Prior studies have demonstrated that each hour of delay in reperfusion results in a substantial decline in functional outcomes, emphasizing the urgent need for timely intervention in AIS management (Fransen et al., 2016). ROC curve analysis further confirmed the prognostic value of baseline NIHSS, serum BNP, OTR, collateral status, and AF in predicting patient outcomes. Serum BNP demonstrated moderate predictive performance based on AUC in the overall cohort. These findings suggest that serum BNP may serve as a useful biomarker for risk stratification and outcome prediction in AIS patients undergoing thrombectomy. However, its clinical application should be interpreted with caution. BNP is not routinely measured in all stroke patients, and its levels can be affected by comorbidities such as congestive heart failure, AF, renal dysfunction, and variations in volume status. As echocardiographic data and standardized assessments of volume status were unavailable in our dataset, we were unable to fully control these potential confounders. Therefore, although BNP may be a useful biomarker for risk stratification, its prognostic utility should be interpreted in the context of a patient’s overall cardiovascular condition. Further prospective studies are warranted to validate its role in routine stroke management.

Absence of AF and shorter OTR were associated with favorable prognosis in the BT group, but these associations were not statistically significant in the DT group. This disparity may reflect underlying hemodynamic differences: AF is frequently associated with impaired cardiac output and reduced cerebral perfusion, which in turn may compromise collateral flow during vessel occlusion (Bao et al., 2024; Su et al., 2017). Moreover, rapid reperfusion confers the greatest clinical benefit when intravenous thrombolysis is effectively administered early, leveraging the thrombolytic sensitivity of fresh clots (Gottlieb et al., 2025; Faizy et al., 2023). Additionally, lower heart rate emerged as a significant prognostic factor in the DT group in univariate logistic analysis, although it did not remain significant in the multivariate model. Emerging evidence suggests that heart rate stability may facilitate hemodynamic balance and improve cerebral perfusion, thereby enhancing recovery potential in AIS. An elevated or unstable heart rate has been associated with poorer outcomes, highlighting the potential prognostic value of heart rate modulation in stroke management (Kallmunzer et al., 2015). A lower heart rate may reflect decreased sympathetic outflow, which has been shown to mitigate secondary brain injury by reducing systemic and cerebral metabolic demands, limiting excitotoxicity, and preserving blood–brain barrier integrity (Maida et al., 2017). Observational data suggest that elevated heart rate is associated with increased post-stroke cardiovascular mortality, indirectly supporting the prognostic relevance of lower baseline heart rate (Heuer et al., 2024). Interestingly, this protective association observed in the DT group was not present in the BT group, potentially reflecting a distinct hemodynamic response in the presence of thrombolytic agents. Such hemodynamic adaptation may help preserve cerebral perfusion without the added influence of thrombolytic-induced reperfusion, thereby reducing oxygen demand and potentially limiting ischemic injury (Sun et al., 2023). Our study contributes to the ongoing debate regarding the optimal thrombectomy strategy for AIS patients with anterior circulation LVO. The absence of significant differences in most outcomes between DT and BT suggests that both approaches are viable, and that treatment selection should be based on individual patient characteristics, particularly collateral status. The increased incidence of hemorrhagic complications in the BT group warrants careful consideration, particularly in patients with poor collateral status or elevated bleeding risk.

Several limitations of this study should be acknowledged. First, the retrospective design may has introduced selection bias and limits the ability to draw causal inferences. Although treatment decisions followed predefined criteria and baseline characteristics were largely comparable, the non-randomized design and clinician discretion may have introduced unmeasured confounding. Additionally, outcome assessments, including the 90-day mRS, were not blinded, potentially introducing observer bias. Second, the study was conducted at a single center, which may affect the generalizability of the findings to other settings with different patient populations and clinical practices. Third, although mCTA is a valuable tool for collateral assessment, it may not reflect dynamic changes in collateral status over time. Additionally, because pre-stroke medication use may not have been consistently or accurately reported by all patients, the potential influence of chronic rate-controlling therapies on baseline heart rate and outcomes could not be excluded. The prognostic value of BNP also must be interpreted with caution, as echocardiographic or volume status data were not available to adjust for potential confounding by cardiac dysfunction or fluid overload. Finally, our predictive models were neither externally validated nor internally calibrated, and should therefore be interpreted as exploratory.

## 5 Conclusion

In conclusion, our findings underscore the critical role of collateral circulation in determining both functional outcomes and complication risk in patients with anterior circulation LVO undergoing endovascular therapy. Although DT and BT yielded broadly similar outcomes, the higher incidence of hemorrhagic complications in the BT group warrants cautious consideration, especially for patients with poor collateral status or increased bleeding risk. These findings emphasize the importance of collateral assessment in guiding treatment decisions and highlight the potential value of serum BNP as a prognostic biomarker. However, further validation in prospective multicenter studies is necessary before its implementation in routine clinical practice, given the selective nature of BNP testing and potential cardiac confounders. Future prospective, multicenter studies are warranted to validate these results and further elucidate the interplay between collateral circulation and thrombectomy strategies.

## Data Availability

The data analyzed in this study is subject to the following licenses/restrictions: the datasets generated and/or analyzed during the current study are available from the corresponding author upon reasonable request. Requests to access these datasets should be directed to Yuan Qin, 13485380083@163.com.

## Supplementary material

SUPPLEMENTARY FIGURE S1 Flow chart of patient enrollment.
