# Supplementary figures and images for: Prognostic impact of collateral circulation in direct thrombectomy versus bridging thrombectomy for acute ischemic stroke patients with anterior circulation large vessel occlusion: a retrospective comparative study
Source: Front Neurosci. 2025 Sep 11;19:1624284. doi: 10.3389/fnins.2025.1624284 (PMC12460346; doi:10.3389/fnins.2025.1624284)

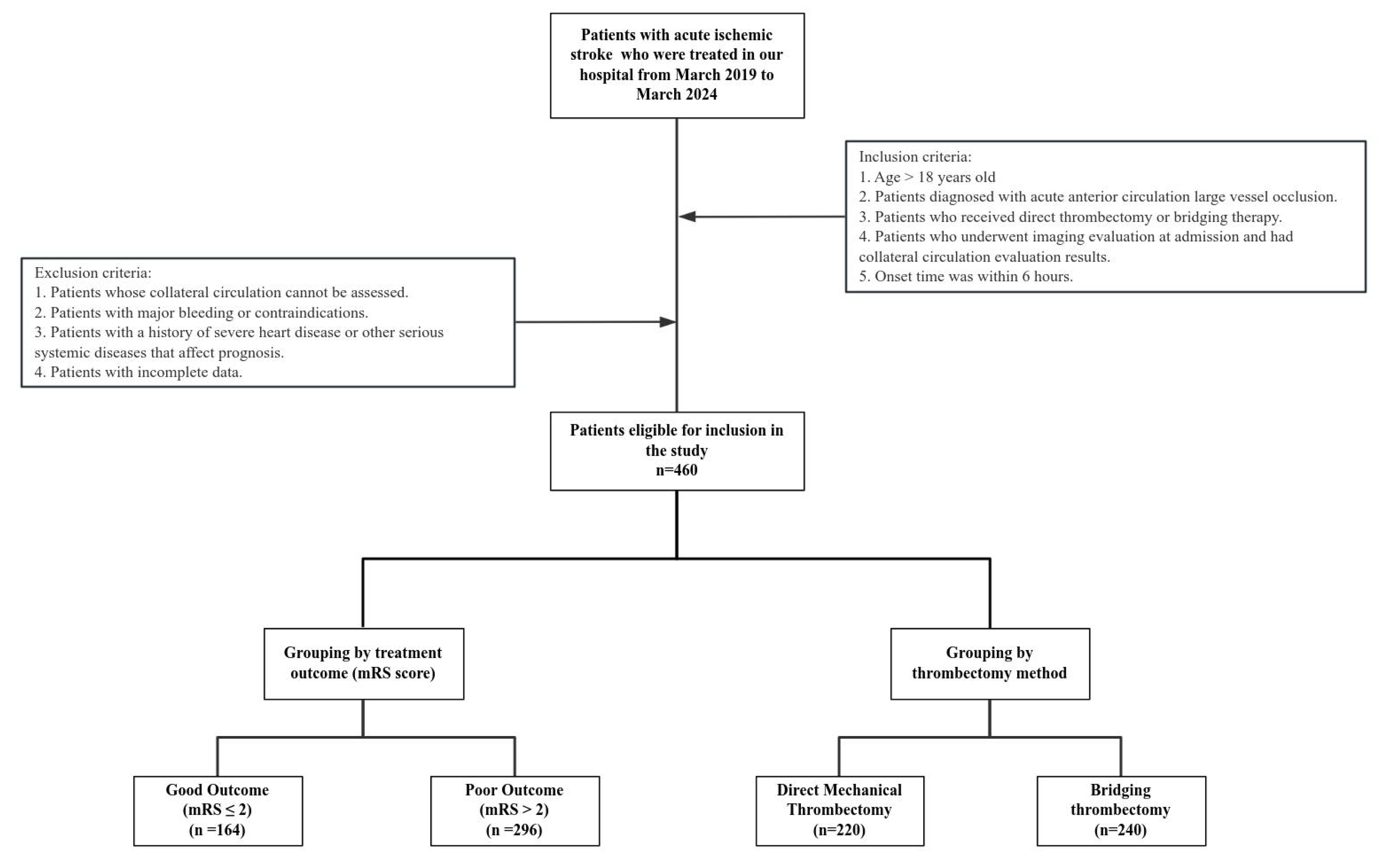

Supplement: SUPPLEMENTARY FIGURE S1 — Flow chart of patient enrollment. [file Image_1.TIF]
