# Supplementary material for: Prognostic impact of collateral circulation in direct thrombectomy versus bridging thrombectomy for acute ischemic stroke patients with anterior circulation large vessel occlusion: a retrospective comparative study
Source: Front Neurosci. 2025 Sep 11;19:1624284. doi: 10.3389/fnins.2025.1624284 (PMC12460346; doi:10.3389/fnins.2025.1624284)
Supplement: Supplementary file 2 [file Table_1.DOCX]

**Table S1. Complete Classification of Adverse Events in All 460 Patients (According to CTCAE v5.0)**

| **Adverse Event Category** | **Specific Event** | **CTCAE ≤2** | **CTCAE >2** |
| --- | --- | --- | --- |
| **Neurological Events** | Asymptomatic intracranial hemorrhage | 66 | 12 |
|  | Symptomatic intracranial hemorrhage (sICH) | - | 38 |
|  | Seizures | 18 | 9 |
|  | Cerebral edema | 10 | 14 |
|  | Reperfusion syndrome | - | 5 |
| **Systemic Events** | Urinary tract infection | 31 | 4 |
|  | Pneumonia (including aspiration) | 42 | 21 |
|  | Acute kidney injury | 26 | 11 |
|  | Cardiac arrhythmia | 34 | 17 |
|  | Myocardial infarction | - | 5 |
|  | Gastrointestinal bleeding | 18 | 12 |
| **Procedure-related Events** | Groin puncture site hematoma | 27 | 6 |
|  | Vessel perforation or dissection | - | 9 |
|  | Contrast agent hypersensitivity | 8 | 1 |
| **Composite Severe Events** | ≥2 concurrent CTCAE Grade ≥3 events | - | 16 |

“-” indicated that the event did not occur in that category or was not applicable.
